# Supplementary figures and images for: Traumatic brain injury reprograms lipid droplet metabolism shaped by aging and diet in Drosophila brain
Source: PLoS One. 2025 Sep 12;20(9):e0332333. doi: 10.1371/journal.pone.0332333 (PMC12431085; doi:10.1371/journal.pone.0332333)

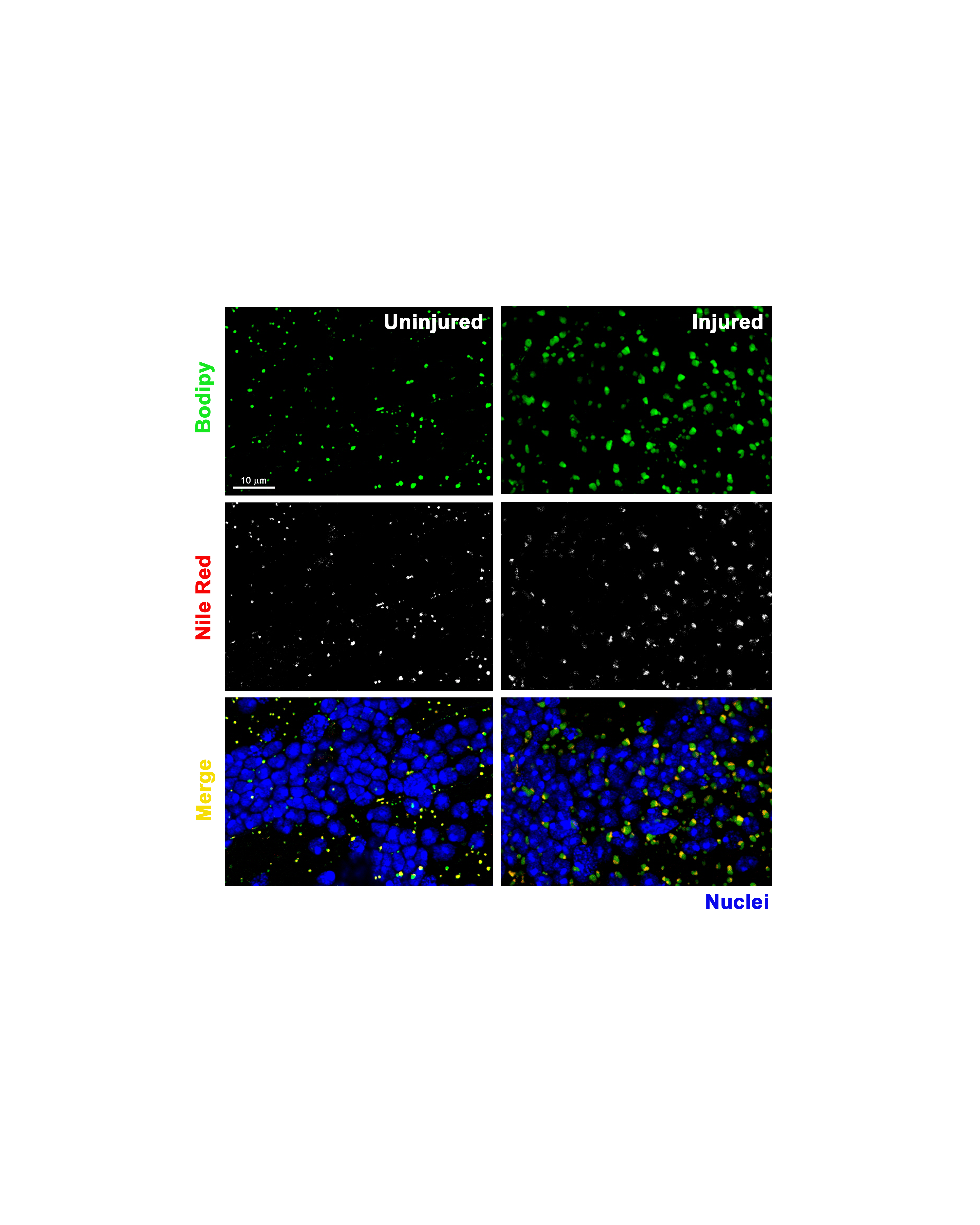

Supplement: S1 Fig — Uninjured (left column) and injured (right column) 8-day old, w1118 flies were stained with Bodipy (top row, green) and Nile Red (middle row, white) in succession, 24 h post-injury. Yellow in the bottom row (merge) indicates overlap of Bodipy and Nile Red signals, and blue indicates DNA (DAPI). (TIF) [file pone.0332333.s001.tif]

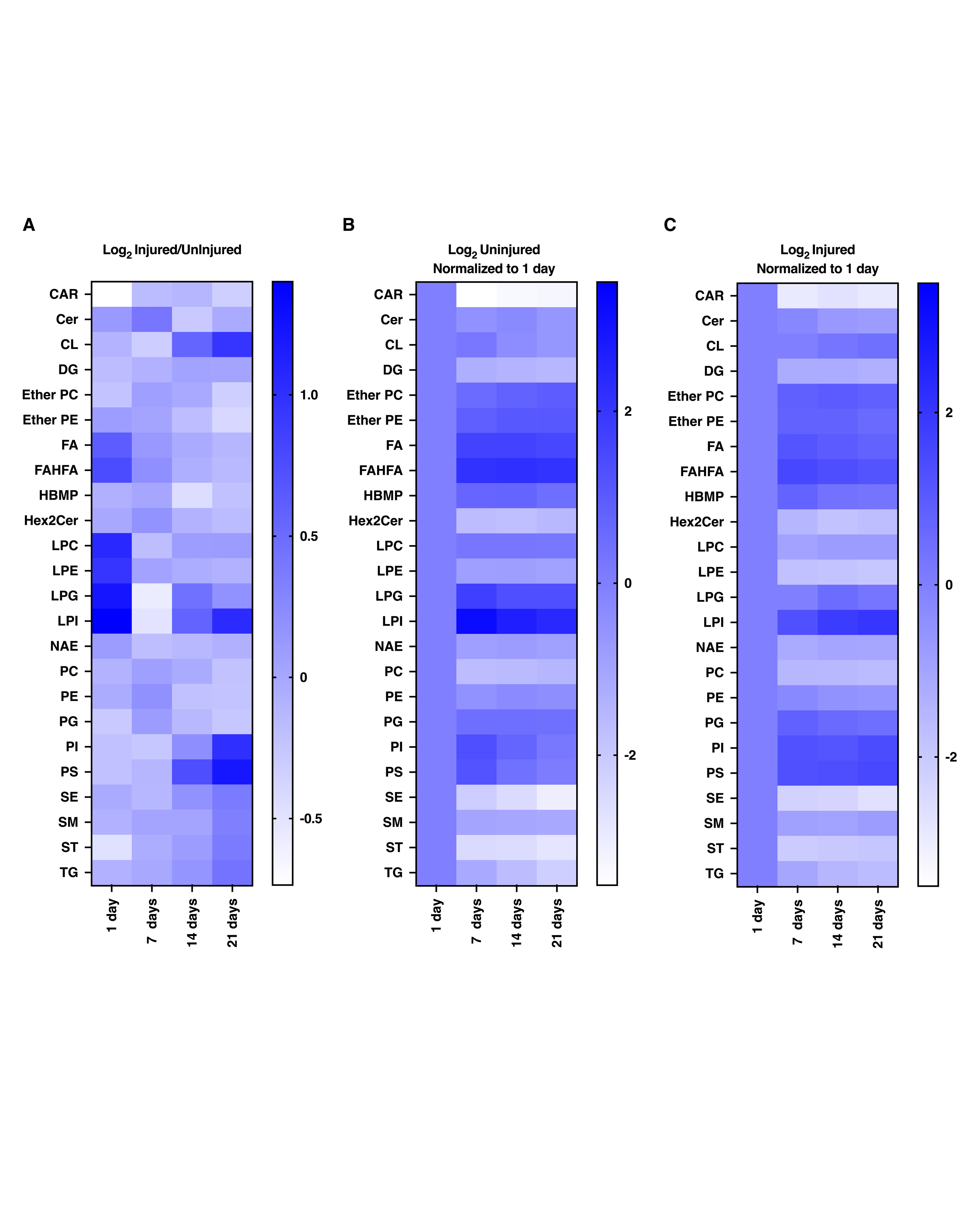

Supplement: S2 Fig — Heat maps of lipid class levels over time after TBI. Lipidomic analysis of heads was performed at 1, 7, 14, and 21 days post-injury of 7-day old, male, w1118 flies. (A) Each box represents the log2-tranformed ratio of the total abundance of all lipid species within a lipid class in injured versus uninjured flies. (B) and (C) Each box shows the log2-transformed ratio of lipid class abundance at 7, 14, and 21 days post-injury, normalized to 1 day levels in (B) uninjured and (C) injured flies. Dark blue is upregulated, and white is downregulated. Abbreviations: acylcarnitine (CAR), ceramide (Cer), cardiolipin (CL), diacylglycerol (DG), ether-linked phosphatidylcholine (Ether PC), ether-linked phosphatidylethanolamine (Ether PE), fatty acid (FA), fatty acid esters of hydroxy fatty acids (FAHFA), hydroxybutyl monophosphate phospholipid (HBMP), dihexosylceramine (Hex2Cer), lysophosphatidylglycerol (LPC), lysophosphatidylethanolamine (LPE), lysophosphatidylglycerol (LPG), lysophosphatidylinositol (LPI), N-acylethanolamine (NAE), phosphatidylcholine (PC), phosphatidylethanolamine (PE), phosphatidylglycerol (PG), phosphatidylinositol (PI), phosphatidylserine (PS), sterol ester (SE), sphingomyelin (SM), and sterol (ST). (JPG) [file pone.0332333.s002.jpg]
